# The Effect of Bovine Serum Albumin on Benzo[a]pyrene Removal by *Lactobacillus* Strains

**DOI:** 10.3390/foods12081676

**Published:** 2023-04-18

**Authors:** Xue Zhang, Zihan Sun, Jinxia Liu, Tao Wang, Bolin Zhang, Hongfei Zhao

**Affiliations:** College of Biological Science & Biotechnology, Beijing Key Laboratory of Forest Food Processing and Safety, Beijing Forestry University, Beijing 100083, China; zhangxue0166@163.com (X.Z.);

**Keywords:** benzo[a]pyrene, *Lactobacillus*, bovine serum albumin, binding mechanism, adsorption kinetics model, adsorption isotherms model

## Abstract

The aim of this study was to investigate the influence of bovine serum albumin (BSA) on the *Lactobacillus*-strain-mediated removal of benzo[a]pyrene (BaP). A combination of 0.5 mg/mL of BSA with 1.0 × 10^10^ CFU/mL bacterial cells had a removal of 49.61% BaP for strain 121, while a combination of 0.4 mg/mL of BSA with 1.0 × 10^10^ CFU/mL bacterial cells had a removal of 66.09% BaP for strain ML32. The results indicated that the binding of BaP to *Lactobacillus*-BSA was stable. BSA maintains *Lactobacillus* activity and BaP removal in the gastrointestinal environment. Heat and ultrasonic treatment of BSA reduced the BaP-binding ability of *Lactobacillus*–BSA. With the addition of BSA, the surface properties of the two strains affected BaP binding. The Fourier-transform infrared (FTIR) data demonstrated that O-H, N-H, C=O, and P=O groups were involved in the binding of BaP to *Lactobacillus*–BSA. Scanning electron microscopy (SEM) results revealed that the morphology of *Lactobacillus*–BSA bound to BaP was maintained. The adsorption of BaP by *Lactobacillus*–BSA was appropriately described by the pseudo-second-order kinetic model and Freundlich isotherm model. BSA enhances the affinity between the bacterial cells and BaP.

## 1. Introduction

Benzo[a]pyrene (BaP) can be detected in fruits, vegetables, cereals, and oils [1,2]. The classification of BaP in food can be categorized into two broad types: endogenous and exogenous, based on their respective origins. Endogenous contamination of BaP occurs during the processing of food through frying, smoking, and baking. The thermal cleavage reactions of lipids, cholesterol, proteins, and carbohydrates generate hydrocarbon radicals that undergo a series of chemical transformations. The combination of hydrocarbon radicals leads to the formation of acetylene, which subsequently undergoes polymerization to form vinylacetylene. The cyclization of the latter produces hexylbenzene, which combines with it to give rise to butylbenzene and tetrahydronaphthalene. Ultimately, these intermediates converge to produce BaP [3,4]. On the other hand, exogenous contamination of BaP arises from industrial activities and the incomplete combustion of coal, oil, natural gas, and other fuels, which generate exhaust gases containing BaP. It can contaminate food through water, air, and soil, ultimately leading to exogenous exposure [5]. BaP has been recognized as the most potent carcinogenic compound that can cause lung, bladder, gastrointestinal, and other types of cancers [6]. Presently, some risk assessments and warning models for BaP indicate that high-priority risk management measures should be implemented [7]. As a result, ascertaining an efficient method for BaP binding is of utmost importance.

*Lactobacillus*, a food-grade-safe microorganism, can be used as a dietary supplement or adsorbent for functional foods. Additionally, *Lactobacillus* could remove toxic substances to reduce damage in vivo and be excreted via feces after being ingested [8,9,10]. The removal of BaP by *Lactobacillus* has become a research priority in recent years. Qi et al. [11] demonstrated that *Lactobacillus plantarum* 121 and *Lactobacillus pentosus* ML32 can adsorb BaP. A previous study by Zhao et al. [12] revealed that *Lactobacillus plantarum* CICC 22135 and *Lactobacillus pentosus* CICC 23163 had high removal rates of BaP from an aqueous medium. Liu et al. [13] reported that simultaneous BaP and *Lactobacillus plantarum* CICC 23121 uptake could dramatically accelerate fecal BaP expulsion and account for BaP detoxification by CICC 23121. Many factors affect the removal of a toxic substance by *Lactobacillus*, one of which is the composition of the food matrix. BaP removal could be affected by the complex and diverse food system. However, protein is an important component of food, and protein-rich meat foods can more easily produce large amounts of BaP [1].

Bovine serum albumin (BSA) is one of the commonly used model proteins for studying the interactions between other substances and proteins [14]. Additionally, this widely used protein can combine with flavonoids, polyphenols, and organic acids [15,16,17], and its binding ability may play a positive role in BaP binding.

The utilization of mathematical models to fit data is a widely carried out and effective approach to investigate adsorption behavior, which involves evaluating the binding strength between the adsorbent and adsorbate and predicting the adsorption capacity of the adsorbent [18]. The adsorption isotherm, which displays the correlation between the concentration of toxic substances and the adsorption capacity, can offer a visual representation of the differences in surface properties of the adsorbent [19]. Adsorption kinetics, on the other hand, can provide valuable information on the adsorption process and the adsorption rate of toxic substances by adsorbents. As such, it is often employed for the optimization and design of adsorption processes [20]. To ascertain the mechanisms of BaP adsorption, various models of adsorption isotherms and kinetics were utilized in this work.

The objectives of this study were to (1) explore the effect of BSA on BaP binding by *Lactobacillus*, (2) investigate the mechanism underlying the binding of BaP by a combination of selected *Lactobacillus* strains and BSA, and (3) examine the kinetics and adsorption isotherms of BaP adsorption by *Lactobacillus* combined with BSA using a mathematical model.

## 2. Materials and Methods

### 2.1. Chemicals and Media

The *Lactobacillus* strains used in this study (*Lactobacillus plantarum* 121 and *Lactobacillus pentosus* ML32) were procured from China Industrial Microbial Culture Preservation Management Center (CICC). The microbes were cultured in the De Man–Rogosa–Sharpe (MRS) medium for 18 h at 37 °C. Subsequently, the viability was evaluated through standard count methods. Standard BaP was obtained from Sigma-Aldrich Co., Ltd. (St. Louis, MO, USA), and other chemicals were purchased from Thermo Fisher Scientific Co., Ltd. (Waltham, MA, USA).

### 2.2. Investigating the Effect of BSA on the Binding of BaP by Lactobacillus

#### 2.2.1. BaP Binding Assay

The mixed solution comprising BaP and BSA was prepared by dissolution in sterilized ultrapure water, in which the BSA concentration was varied in incremental steps of 0.1 mg/mL, ranging from 0 to 0.5 mg/mL, and the BaP concentration was 10 μg/mL.

To separate the cells of *Lactobacillus* strains from the MRS medium, the incubation tubes containing the cell pellets were centrifuged at 8000× *g* for 10 min at 4 °C, and were subsequently subjected to two washes with sterilized saline water. The culture (approx. 1.0 × 10^10^ CFU/mL) was suspended in a 1.0 mL mixed solution, and the mixture was then incubated for 4 h at 37 °C. Following incubation, chloroform (500 μL) was added. To extract the supernatant, the cells were isolated by centrifugation (at 8000× *g* for 10 min at 4 °C).

The BaP concentration of the collected supernatant was determined using high-performance liquid chromatography (HPLC) (LC-20AT; Shimadzu Co., Ltd., Nagoya, Japan) equipped with an InertSustain-C18 column (4.6 × 250 mm; 5-Micron) and a RID-20 differential detector. Each sample was subjected to filtration through a 0.22 μm pore membrane. Specifically, 20 μL of each sample was injected into the HPLC system to quantify the BaP concentration. The mobile phases were methanol with a flow rate of 1 mL/min at 40 °C. The detection wavelength was 290 nm. It was quantified using a calibration curve.

A positive control consisting of sterile H_2_O and BaP was utilized in all the experiments. The BaP binding percentage was calculated using the following equation:

BaP binding percentage (%) = (1 − BaP peak area of sample/BaP peak area of positive control) × 100%(1)

#### 2.2.2. Stability of *Lactobacillus*–BSA Binding to BaP

The stability of BaP binding to *Lactobacillus*–BSA was determined by elution using benzene, the PBS buffer, and ethanol (55%, *v*/*v*). The biomass was centrifuged, and then vortexed using benzene, the PBS buffer, and ethanol benzene three times for 5 min. The mixture was then centrifuged. The supernatant was collected and analyzed by HPLC.

#### 2.2.3. Effect of BaP Binding in a Simulated Gastrointestinal Environment

For the simulated gastric juice, 16.4 mL HCl (1 mol/L) and 10 g pepsin were added to 800 mL of the PBS buffer. Subsequently, the PBS buffer was added to reach a final volume of 1 L and the pH was adjusted to 2.0–3.0 (8 mol/L HCl). Following the preparation of the mixture, it underwent filtration using a 0.22 μm sterile filter membrane. For the stimulated intestinal juice, pancreatic juice was composed of 0.1% trypsin, 1.1% NaHCO_3_, and 0.2% NaCl with the pH adjusted to 8.0 (1 mol/L NaOH). The 0.9% bile solution salt was adjusted to pH 8.0. The mixture was composed of pancreatic juice and bile juice in a 2:1 ratio. Ultimately, the mixture underwent filtration using a 0.22 μm sterile filter membrane.

To explore the tolerance of strains in in vitro simulation studies in the presence of BSA, the culture was suspended in a simulated gastric juice solution, in which the cell concentration was 1.0 × 10^10^ CFU/mL and the BSA concentration was adjusted to 0.5 mg/mL in strain 121 and 0.4 mg/mL in strain ML32. BSA was not used in the control group. The incubation of the mixture was carried out for 2 h at 37 °C, followed by the determination of the total viable count using plate count methods. Subsequently, 1 mL of the mixture was added into 1 mL of the simulated intestinal juice and incubated for 2 h at 37 °C. Following incubation, the total viable count was determined using plate count methods. The survival rate (%) was calculated as follows:

Survival rate (%) = *C*/*C*_0_ × 100% (2)

where *C* is the total viable cell count in the simulated gastrointestinal environment and *C*_0_ is the total viable cell count without simulated gastrointestinal environment treatment.

To explore the effect of BaP binding by *Lactobacillus*–BSA under a simulated gastrointestinal environment, the culture was suspended in the simulated gastric juice solution, in which the cell concentration was 1.0 × 10^10^ CFU/mL, the BaP concentration was 10 μg/mL, and the BSA concentration was adjusted to 0.5 mg/mL in strain 121 and 0.4 mg/mL in strain ML32. BSA was not used in the control group. The mixture was incubated for 2 h at 37 °C; the supernatants were collected for the analysis of the residual BaP concentration by HPLC. The residual BaP concentration was then determined using HPLC after 1 mL of the mixture was added to 1 mL of the simulated intestinal juice (BaP 10 μg/mL) and incubated for 2 h at 37 °C.

#### 2.2.4. Heat- and Ultrasonically Treated BSA’s Impact on the Binding of BaP by *Lactobacillus*–BSA

To determine the effect of heat treating BSA on the binding of BaP by *Lactobacillus*-BSA, BSA solution was heated for 10 min at 25 ℃, 37 °C, 50 °C, and 80 °C. To assess the effect of ultrasonic treatment of BSA on the binding of BaP by *Lactobacillus*–BSA, the cells were subjected to ultrasound at 25 °C for 2 min, 5 min, 10 min, 15 min, and 20 min. Following incubation for 4 h at 37 °C, the samples were analyzed by HPLC.

### 2.3. Exploring the Mechanism of BaP Binding to Lactobacillus–BSA

#### 2.3.1. Effect of BSA on the Properties of Bacterial Cell Surface

##### Surface Hydrophobicity

The culture (approx. 1.0 × 10^10^ CFU/mL) was suspended in 1.0 mL of the BSA solution with varying concentrations of 0, 0.1, 0.2, 0.3, 0.4, and 0.5 mg/mL. The mixture was incubated for 4 h at 37 °C. The cells were subsequently collected by centrifugation (8000× *g*, 10 min, 4 °C), followed by triple washing with distilled water, and adjusted to an OD of 0.5–0.6 at 600 nm (*H*_0_). Following that, a 4.5 mL cell suspension was mixed with 1.5 mL of xylene and vortexed for 2 min. The mixture was subjected to phase stabilization and separation for 30 min at 25 °C, following which the OD of the aqueous phase was measured at 600 nm (*H*_1_). The hydrophobicity value (%) was calculated according to:

H (%) = ((*H*_0_ − *H*_1_)/*H*_0_) × 100% (3)

The Pearson correlation coefficient analysis was used to determine the correlation between the surface hydrophobicity and BaP binding percentage of the strains using the Statistical Package for Social Science (SPSS) (version 25.0) software.

##### Automatic Aggregation

Based on the methodology in Section Surface Hydrophobicity, the culture (approx. 1.0 × 10^10^ CFU/mL) was suspended, incubated and adjusted to an OD of 0.5–0.6 at 600 nm (*A*_0_). The cell suspension was allowed to stand for 4 h and the absorbance of the supernatant was measured at 600 nm (*A*_1_). The automatic aggregation value (%) was calculated as follows:

A (%) = (1 − *A*_1_/*A*_0_) × 100% (4)

The correlation between the automatic aggregation and BaP binding percentage of the strains was determined using the SPSS (version 25.0) software to perform the Pearson correlation coefficient analysis.

#### 2.3.2. FTIR Analysis

To discern the potential functional groups and binding sites involved in the binding of BaP by *Lactobacillus*–BSA, powdered samples (2 mg) were mixed with 200 mg of (spectral) KBr and ground using an agate mortar. The analysis was performed using an X70 FTIR spectrophotometer (Netzsch Co., Ltd., Selb, Germany). The FT-IR spectra were scanned at 25 °C, with a spectral range of 4000–400 cm^−1^.

#### 2.3.3. SEM Analysis

To characterize the morphology of bacterial cells using scanning electron microscopy (SEM), the cells were pre-fixed (at 4 °C, in 2.5% glutaraldehyde), dehydrated (for 10 min, with 30–90% gradient ethanol) and freeze-dried. The samples underwent the procedures involving gold sputtering, observation, and photography via an scanning electron microscope (ZEISS Gemini 300, Carl Zeiss Co., Ltd., Oberkochen, Germany).

#### 2.3.4. Isotherm Model Studies

The relationship between the mass of BaP adsorbed per unit mass of adsorbent and the aqueous-phase BaP concentration at equilibrium and a constant temperature is described by the adsorption isotherm. The cells of *Lactobacillus* strains were suspended in different BaP concentrations (2, 5, 10, 20, 40, 60, and 80 μg/mL), and the concentration of cells was adjusted to 1 g/L. Furthermore, in the *Lactobacillus*–BSA group, the concentration of BSA was adjusted to 0.5 mg/mL and 0.4 mg/mL for strains 121 and ML32, respectively. The tests were conducted at 37 °C for 4 h.

The Langmuir isotherm was employed for the adsorption of BaP:

(5) $$q_{e}=q_{max}\times K_{L}\times C_{e}{\times (1+K_{L}\times C_{e})}^{-1}$$

where *q_e_*, *q_max_*, *C_e_*, and *K_L_* are the amounts of BaP per unit weight of the adsorbent at the adsorption equilibrium (mg/g), maximum BaP adsorption capacity (mg/g), BaP concentration in solution at equilibrium (mg/L), and the Langmuir adsorption constant (L/mg), respectively.

To illustrate the Langmuir isotherm accurately, another dimensionless constant known as a separation factor (*R_L_*) can be defined as follows:

(6) $$R_{L}={\left(1+K_{L}\times C_{0}\right)}^{-1}$$

where *C*_0_ is the initial BaP concentration (2, 5, 10, 20, 40, 60, and 80 μg/mL).

The Freundlich isotherm is used to describe the adsorption of a broad range of adsorbates, as shown in the following formula:

(7) $$q_{e}=K_{F}\times C_{e}{}^{1/n}$$

where *K_F_* is the Freundlich constant related to adsorption capacity [(mg/g)/(mg/L)^n^] and *n* is the adsorption intensity constant.

#### 2.3.5. Adsorption Kinetics Studies

For adsorption kinetics studies, the *Lactobacillus* cells were suspended in BaP concentrations (10 μg/mL), and the cell concentration was adjusted to 1 g/L. Additionally, in the *Lactobacillus*–BSA group, the concentration of BSA was adjusted to 0.5 mg/mL and 0.4 mg/mL for strains 121 and ML32, respectively. The tests were run at 37 °C and analyzed at 2, 5, 10, 15, 20, 25, 30, and 45 min. The pseudo-first-order model, pseudo-second-order model, Elovich model and Weber–Morris model were used for determining BaP adsorption.

Pseudo-first-order kinetic rate equation:

(8) $$\mathrm{In}\left(q_{e}\cdot q_{t}\right)=\mathrm{In}q_{e}\cdot K_{1}\cdot t$$

where *q_t_* is the concentration of adsorbed BaP per unit weight of the adsorbent at a given time (*t*) (mg/g). *K*_1_ is the pseudo-first-order kinetic rate constant (min^−1^).

Pseudo-second-order kinetic rate equation:

(9) $$t\times {q_{t}}^{-1}={\left(K_{2}\times {q_{e}}^{\wedge 2}\right)}^{-1}+t\times {q_{e}}^{-1}$$

where *K*_2_ is the pseudo-second-order kinetic rate constant (g mg^−1^ min^−1^).

The following equation describes the Elovich model:

(10) $$q_{t}=a+b\cdot \mathrm{In}t$$

where *a* (mg g^−1^ min^−1^) and *b* (g mg^−1^) are the Elovich model parameters.

The following equation describes the Weber–Morris model:

(11) $$q_{t}=K_{p}\cdot t^{1/2}+C$$

where *K_p_* is the intra-particle diffusion rate constant (mg g^−1^ min^−1/2^). *C* is the constant related to the boundary layer thickness (mg g^−1^).

### 2.4. Statistical Analysis

All experiments and analyses were performed at least three times. All data were expressed as mean ± standard deviation. Data analysis was performed using SPSS (version 25.0) and Origin (version 8.0). The statistical analysis was carried out using the one-way analysis of variance (ANOVA).

## 3. Results

### 3.1. The Effect of BSA on the Binding of BaP by Lactobacillus

#### 3.1.1. The BaP-Binding Ability of *Lactobacillus*–BSA

**Figure 1:**
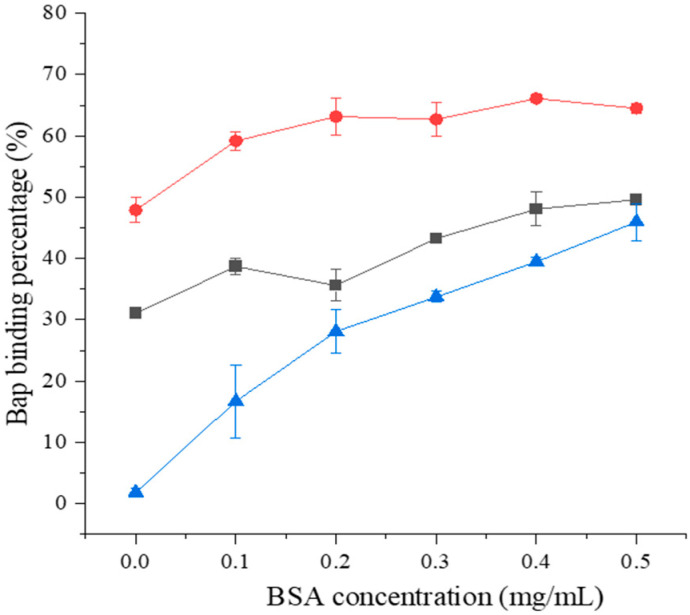
The BaP-binding ability of *Lactobacillus*–BSA. 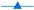 Control, 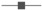 121, and 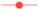 ML32.

The BaP binding percentage of BSA alone was lower than that of the combination of BSA with bacterial cells (Figure 1). Moreover, the BaP binding percentage of *Lactobacillus*–BSA increased with increasing BSA concentrations for both strains. *L. plantarum* 121–BSA could bind 31.06–49.61% of the BaP, whereas *L. pentosus* ML32–BSA could bind 47.91–66.09% of the BaP. For *L. plantarum* 121, the maximum BaP binding percentage was achieved with 0.5 mg/mL of BSA, while for *L. pentosus* ML32, the maximum BaP binding percentage was achieved with 0.4 mg/mL of BSA. As a result, these effective BSA concentrations were selected for further studies.

#### 3.1.2. Stability of BaP Binding to *Lactobacillus*–BSA

**Figure 2:**
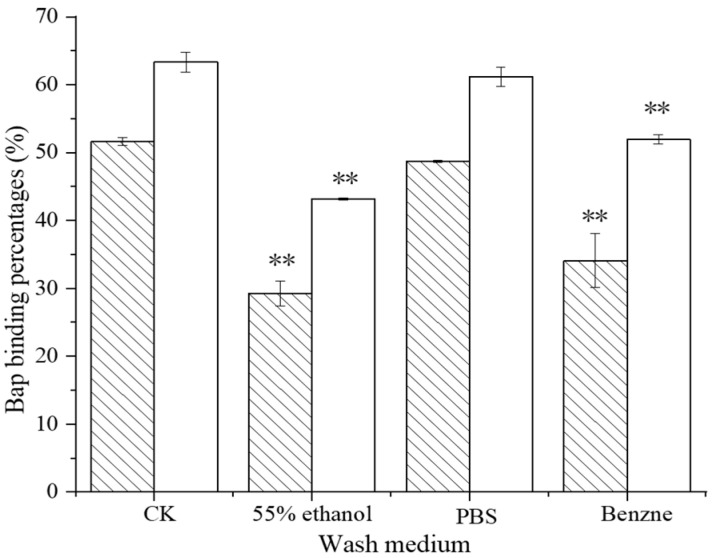
The stability of the binding of *Lactobacillus*–BSA to BaP. Note: ** *p* < 0.01. 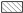 121 and 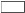 ML32.

Triple washing of the complex with the PBS buffer led to the reduction of 2.93% and 2.30% in the BaP binding percentage (Figure 2). More BaP was released using 55% ethanol and benzene. The reductions of 22.38% (*L. plantarum* 121-BSA) and 20.35% (*L. pentosus* ML32–BSA) in the BaP–binding percentage were observed with 55% ethanol and 17.43% (*L. plantarum* 121-BSA) and 11.33% (*L. pentosus* ML32-BSA) benzene (*p* < 0.01). In general, organic solvents have a greater effect on the stability of BaP in binding to *Lactobacillus*–BSA.

#### 3.1.3. Simulated Gastrointestinal Tract Analysis

This study used a model to simulate the human gastrointestinal tract and investigate the survival rate of bacterial cells in the presence of BSA as well as the percentage of BaP bound by *Lactobacillus*–BSA.

Both the strains exhibited high survival rates in the simulated gastrointestinal tract in the presence of BSA (Table 1). The survival rates in the simulated gastric juice and intestinal juice for *L. plantarum* 121 were 69.30% and 14.47%, respectively, while they were 80.12% and 13.20%, respectively, for *L. pentosus* ML32. This indiates that BSA has a protective effect on bacterial cells. Furthermore, for both strains, the survival rate was observed to be higher in the simulated gastric juice compared to thr simulated intestinal juice.

*Lactobacillus*–BSA demonstrated strong binding to BaP in the simulated gastrointestinal tract (Table 2). Similarly, the BaP binding percentage of simulated gastric juice was higher than that of the simulated intestinal juice for both strains. For *L. plantarum* 121, the BaP binding percentages of *Lactobacillus*–BSA in the simulated gastric juice and intestinal juice were 74.17% and 6.33%, respectively. For *L. pentosus* ML32, the BaP binding percentages of *Lactobacillus*–BSA in the simulated gastric juice and intestinal juice were 72.86% and 16.88%, respectively. In the simulated gastrointestinal tract, the BaP binding percentage of bacterial cells increased when combined with BSA (*p* < 0.05).

#### 3.1.4. The Effect of Heat- and Ultrasonically Treated BSA on the Binding of *Lactobacillus*–BSA to BaP

**Figure 3:**
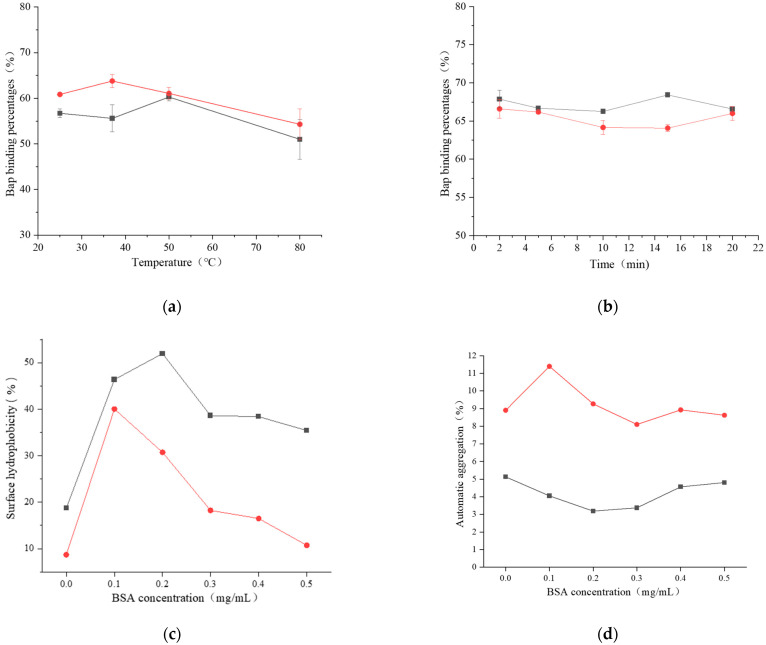
The effect of heat- (**a**) and ultrasonically (**b**) treated BSA on the binding of *Lactobacillus*–BSA to BaP. The effect of BSA on surface hydrophobicity (**c**) and automatic aggregation (**d**) of strains. 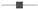 121; 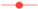 ML32.

The BaP-binding ability of *L. plantarum* 121–BSA was almost unaffected by heat treatment (Figure 3a). However, there was a reduction in the percentage of BaP binding by *L. pentosus* ML32–BSA at 80 °C; it was only 54.30%. The BaP binding percentage of *Lactobacillus*–BSA tended to decline between 2 and 10 min (Figure 3b). However, when the ultrasonic time increased to above 10 min, the BaP binding percentage of *L. plantarum* 121–BSA increased at first and then decreased to 66.58%, whereas that of *L. pentosus* ML32–BSA did not significantly change. These findings indicate that heat and ultrasonic treatment with BSA adversely affect the binding of BaP to *Lactobacillus*–BSA.

### 3.2. The Mechanism of BaP Binding to Lactobacillus–BSA

#### 3.2.1. The Effect of BSA on Bacterial Cell Surface Properties

The surface hydrophobicity initially increased and then decreased as the BSA concentration increased for the two strains (Figure 3c). Notably, the maximum surface hydrophobicity values were found at 0.2 mg/mL and 0.1 mg/mL, which were 51.97% (*L. plantarum* 121) and 40.04% (*L. pentosus* ML32), respectively. Overall, BSA improved the hydrophobicity of the two *Lactobacillus* strains in the concentration range of 0–0.5 mg/mL. As presented in Figure 3d, the automatic aggregation of *L. plantarum* 121 first decreased and then increased as the BSA concentration increased, which was contrary to the change observed in *L. pentosus* ML32. The minimum automatic aggregation value for *L. plantarum* 121 was found to be 3.18% at a concentration of 0.2 mg/mL. The maximum automatic aggregation value for *L. pentosus* ML32 was 11.40% and was discovered at 0.1 mg/mL. These data indicate that BSA had no negative effect on the automatic aggregation of the two *Lactobacillus* strains.

The absolute values of the Pearson correlation coefficient of the surface hydrophobicity of the two strains and the BaP binding percentage were 0.19 and 0.22, while they were 0.09 and 0.13 for the automatic aggregation of the two strains and the BaP binding percentage (Table 3). In the presence of BSA, a weak correlation was observed between the surface properties of the strains and the BaP binding percentage.

#### 3.2.2. FTIR Analysis

**Figure 4:**
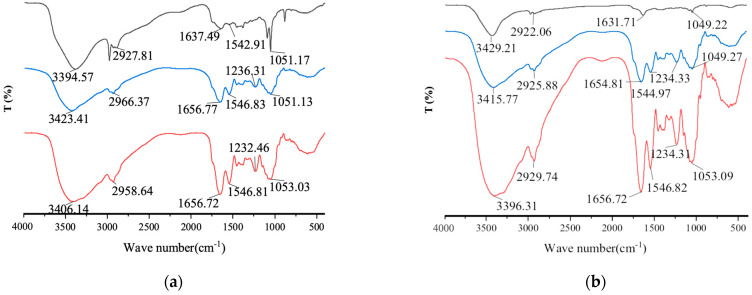
The FTIR spectrum in the mid-infrared region (4000 ~ 500 cm^−1^), (**a**) 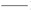 121, 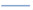 121 + BaP + BSA, 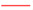 121 + BaP; (**b**) 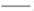 ML32, 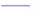 ML32 + BaP + BSA, and 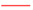 ML32 + BaP.

FTIR spectroscopy was utilized as a method for identifying the possible functional groups and binding sites that may be involved in BaP binding. The average FTIR spectra of the *Lactobacillus*–BSA–BaP interaction are shown in Figure 4a,b. It was observed that the wavelengths were in the range of 3500–3200 cm^−1^ for the two strains, which could be attributed to the N-H and O-H characteristic vibration absorption peaks. The -CH stretching of the methyl and methylene groups was observed in the wavelength range 2970–2840 cm^−1^, which could be attributed to the fatty acids of the membrane phospholipids participating in the binding process. The peak of amide I shifted by 19 and 25 cm^−1^ after *Lactobacillus*–BSA binding to BaP, which could be attributed to the changes in C=O in amides. The intensity of amide II (1650–1530 cm^−1^) was substantially weakened after adsorption, indicating that the functional groups of proteins participated in the binding process. The peak of P=O (lipid and polysaccharide) was near 1240 cm^−1^, and the intensity of the absorption peak was weakened following the binding. The peak near 1050 cm^−1^ was the result of the combined action of stretching of the phosphoric acid group, sugar hydroxyl group, amide V, and S=O, which shifted when *Lactobacillus*–BSA was bound to BaP.

#### 3.2.3. SEM Analysis

**Figure 5:**
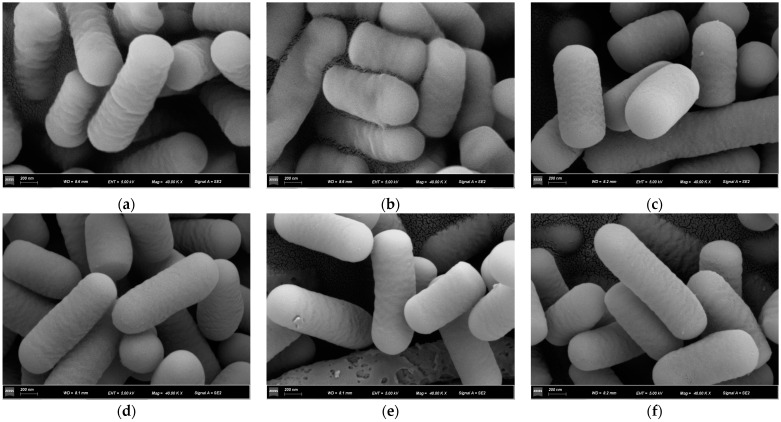
The SEM image of the strains (40,000 magnification), (**a**,**b**)—strain 121 before and after binding BaP, (**c**)—strain 121 combined with BSA binding BaP, (**d**,**e**)—strain ML32 before and after binding BaP, (**f**)—strain ML32 combined with BSA binding BaP.

SEM was used to confirm the morphology of the BaP-exposed and -unexposed bacterial cells. An alteration in the binding of *Lactobacillus*–BSA to BaP was observed (Figure 5). The surface morphology of the bacterial cells that were not exposed to BaP was observed to be free from disruptions, appearing smooth. Compared with the unexposed bacterial cell (control), the morphology of BaP-exposed cells was distinctly deformed and damaged. However, the cell surface of *Lactobacillus*–BSA bound to BaP was smooth and full.

#### 3.2.4. Biosorption Isotherm of BaP

**Figure 6:**
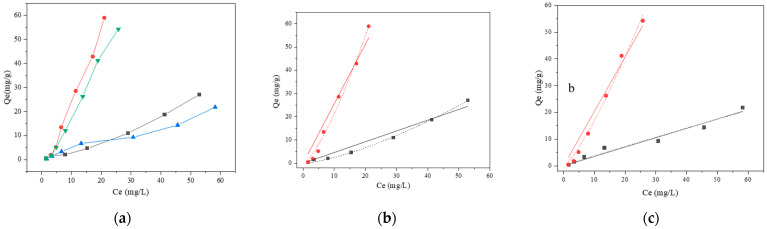
(**a**) Adsorption isotherms of BaP; 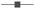 121, 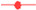 121 + BSA, 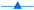 ML32, and 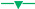 ML32 + BSA. (**b**) Isotherm models of BaP; 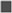 121, 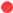 121 + BSA, 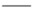 Langmuir, 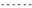 Freundlich, 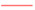 Langmuir, and 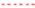 Freundlich. (**c**) Isotherm models of BaP; 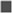 ML32, 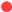 ML32 + BSA, 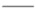 Langmuir, 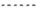 Freundlich, 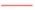 Langmuir, and 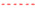 Freundlich.

An adsorption equilibrium is reached when the concentration of adsorbed BaP is equal to the concentration of desorbed BaP, and the equilibrium value in the solution is constant. An increase in the quantity of BaP per unit weight of the adsorbent at the adsorption equilibrium was observed for *Lactobacillus*–BSA when compared with the *Lactobacillus* control (Figure 6a). In this study, two isotherm models (Langmuir and Freundlich) were used to predict the adsorption model of BaP. Figure 6b,c demonstrates Langmuir and Freundlich isothermal adsorption model fitting results. The nature of the isotherm can be determined based on the value of *R_L_*, where *R_L_* = 0 represents irreversibility, *R_L_* = 1 represents linearity, *R_L_* between 0 and 1 represents favorableness, and *R_L_* > 1 represents unfavorableness. The calculated *R_L_* values of *L. plantarum* 121, *L. pentosus* ML32, *L. plantarum* 121–BSA, and *L. pentosus* ML32–BSA were all in the range of 0–1, signifying favorable adsorption. Based on the *R^2^* values, the Freundlich isotherm model is an excellent fit for BaP adsorption by *Lactobacillus*–BSA, with high correlation coefficients of *R*^2^ = 0.9911 for *L. plantarum* 121–BSA and *R*^2^ = 0.9905 for *L. pentosus* ML32–BSA (Table 4). Additionally, as shown in Table 4, the affinity of the complex could be measured by the coefficient *K_F_* in the Freundlich isotherm model. In the presence of BSA, the *K_F_* values of *L. plantarum* 121 changed from 0.0919 to 0.9182, whereas those of *L. pentosus* ML32 improved from 0.4125 to 0.9074. Additionally, the value of n indicates that adsorption is poor, relatively difficult or excellent when *n* < 1, 1 < *n* < 2 and 2 < *n* < 10, respectively. The n values of all BaP adsorption processes by *L. plantarum* 121, *L. pentosus* ML32, *L. plantarum* 121–BSA, and *L. pentosus* ML32–BSA were in the range of 1–2, or *n* < 1.

#### 3.2.5. Kinetic Model Studies

**Figure 7:**
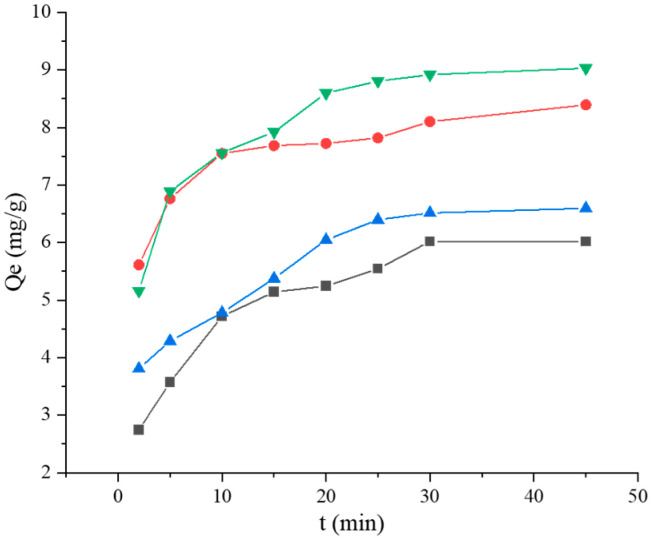
The adsorption kinetics curve of BaP. 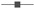 121, 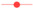 121 + BSA, 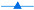 ML32, and 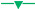 ML32 + BSA.

**Figure 8:**
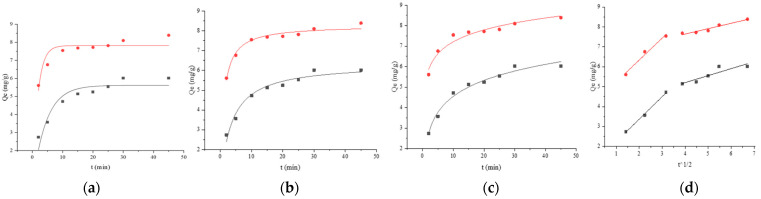

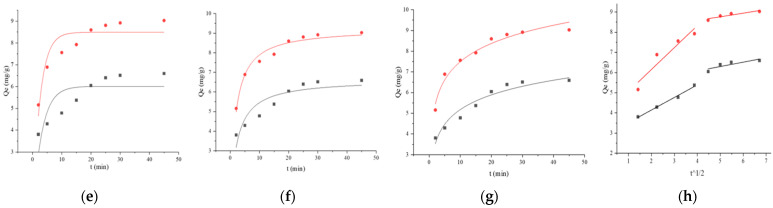
Adsorption kinetic models fitting of BaP by strain 121 and its combination with BSA; 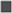 121, 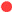 121 + BSA, (**a**) pseudo-first-order, (**b**) pseudo-second-order, (**c**) Elovich, and (**d**) Weber–Morris. Adsorption kinetics models fitting of BaP by strain ML32 and its combination with BSA; 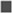 ML32, 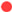 ML32 + BSA, (**e**) pseudo-first-order, (**f**) pseudo-second-order, (**g**) Elovich, and (**h**) Weber–Morris.

The time profile of the BaP adsorption is shown in Figure 7. The BaP adsorption capacities of *L. plantarum* 121, *L. pentosus* ML32, *L. plantarum* 121–BSA, and *L. pentosus* ML32–BSA increased rapidly before 15 min and reached the peak at approximately 30 min; more binding sites increased the likelihood of binding occurring at the start. In this study, four models (pseudo-first-order kinetic, pseudo-second-order kinetic, Elovich, and Weber–Morris models) were used to predict BaP adsorption (Figure 8). According to the correlation coefficient values (*R*^2^) from the linear regression analysis for the pseudo-first-order kinetic, pseudo-second-order kinetic, and Elovich models (Table 5), the kinetic models of *Lactobacillus* binding BaP fit with the Elovich kinetic model; the *R*^2^ value was 0.9793 for *L. plantarum* 121 and 0.9417 for *L. pentosus* ML32. The results obtained in the presence of BSA clearly fit the pseudo-second-order kinetic model. For *L. plantarum* 121–BSA, the *R*^2^ value was 0.9681, while for *L. pentosus* ML32–BSA, the *R*^2^ value was larger (0.9708). Furthermore, the calculated adsorption capacity values were in agreement with the experimental values (Qexp) (Table 5). In addition, for the rate parameter *K*_2_ in the pseudo-second-order kinetic model, the value for *L. plantarum* 121–BSA was greater than that for *L. pentosus* ML32–BSA. Based on the results of the Weber–Morris model (Figure 8d,h), the two straight lines did not pass through the origin, and the plot was divided into two stages.

## 4. Discussion

BaP is recognized as a strong carcinogen in food production and processing, which threatens human health [6]. The removal of BaP holds considerable importance in the context of ensuring food safety. Studies have shown that *L. plantarum* 121 and *L. pentosus* ML32 can adsorb BaP [11]. This is the first study to explore the effects of BSA on the binding of BaP to *Lactobacillus*.

BSA has different binding effects on various substances, which provides an important basis for its role in the body [21,22]. In this study, *Lactobacillus*–BSA significantly improved the BaP binding percentage. All results indicated that BSA itself had the ability to bind to BaP; however, it was not as strong as that of *Lactobacillus*–BSA. This revealed that BaP binding results from the synergistic effects of *Lactobacillus* and BSA.

According to these data, the binding of the toxin to the biomass is not very stable. If binding is weak, the toxin would be released by continuous washing of the complex. The findings reveal that washing the complex with benzene, ethanol, and PBS buffer all led to the desorption of BaP. However, the binding of BaP to *Lactobacillus*–BSA still maintained certain stability. This indicates that the BaP bound by *Lactobacillus*–BSA was not easily released, which would be highly desirable.

BaP bioaccessibility is a major aspect of BaP control, and the stability of BaP binding in the gastrointestinal environment is critical [23]. To investigate the effect of BaP binding to *Lactobacillus*–BSA within the gastrointestinal tract, an in vitro simulation model was used. The in vitro simulation showed that supplementation with BSA maintained the survival rate of bacteria, indicating that BSA has a protective effect on *Lactobacillus*. Similarly, a rise in the BaP binding percentage was observed in the two strains in combination with BSA. However, the BaP binding percentage in the simulated intestinal juice was found to be significantly lower than that observed in the simulated gastric juice. Previous studies have indicated that the properties of bacterial cells and proteins change greatly after the two stages of gastrointestinal digestion [24].

Heat treatment can cause structural changes in proteins, protein denaturation, and the formation of thermal polymers [25]. Additionally, superheat treatment can change the structural properties of BSA and reduce the exposure of its hydrophobic groups [26]. Ultrasonic treatment can alter the secondary structure of proteins, in addition to changing their hydrophobic properties [27]. As a result, a decrease in the number of binding sites of BSA for BaP resulted in a reduction in the BaP binding percentage.

Previous studies have confirmed that the hydrophobicity and automatic aggregation of bacterial cells are related to adhesion properties, which are important prerequisites for functioning in the human body [28]. Various pretreatments expose the hydrophobic groups on the bacterial surface, enhancing the binding of BaP [29]. Zhao et al. [30] indicated that hydrophobic interactions affect the removal of di-*n*-butyl phthalate (DBP) by LAB and that a decrease in the DBP adsorption rate can be attributed to a reduction in the number of hydrogen bonds and electrostatic interactions. In this study, BSA improved the surface hydrophobicity of the two strains, which could be attributed to the enhancement of surface hydrophobicity that increased the BaP binding percentage. The correlation between the BaP binding percentage and the surface properties of the selected *Lactobacillus* strains was analyzed using the Pearson correlation coefficient. The greater the absolute value of the correlation coefficient, the stronger the correlation. The closer the correlation coefficient is to 1 or −1, the stronger was the correlation, and the closer the correlation coefficient is to 0, the weaker was the correlation. A weak correlation between the surface properties of the two *Lactobacillus* strains and the BaP binding percentage in the presence of BSA was noted.

By analyzing the FTIR spectrum, it can be assumed that some functional groups of polysaccharides, lipids, and proteins are involved in BaP binding. Shen et al. [31] demonstrated that peptidoglycans and proteins on cell walls are the major components involved in AA adsorption. Ge et al. [32] revealed that the C-O, O-H, and N-H groups related to proteins and peptidoglycans play a major role in tenuazonic acid adsorption. SEM could explore the relationship between the changes in cell surface structure and BaP binding by *Lactobacillus*–BSA. The SEM results showed that the morphology of *Lactobacillus*–BSA did not change significantly after the adsorption of BaP. BSA does not change the morphology of bacterial cells.

In this study, Langmuir and Freundlich isotherm models were used to predict the adsorption of BaP by *Lactobacillus*–BSA. The corresponding fitting parameters were obtained by fitting and analyzing the adsorption isotherms. These adsorption isotherm parameters reflect the affinities of the binding sites and binding mechanisms [33]. According to the correlation coefficient results, the Freundlich model provided a better description of the kinetics of BaP adsorption by *Lactobacillus*–BSA than the Langmuir model did. The basis for this is the supposition that BaP adsorption is probably heterogeneous between layers. *K_F_* reflects the affinity between the adsorbent and adsorbate. The larger the value of *K_F_*, the easier the adsorption of the adsorbate [34]. An increase in *K_F_* values was observed for BaP adsorption by *Lactobacillus*–BSA compared to *Lactobacillus*, which indicated that the supplementation of BSA enhanced the affinity between bacterial cells and BaP. Another parameter (1/*n*) was used to describe the adsorption intensity, with a value of 1/*n* between 0.1 and 0.5, indicating that adsorption is easy to conduct [35]. Based on these results, the binding process of BaP to *Lactobacillus*–BSA was not optimal.

According to the kinetic model studies, the kinetic models of *Lactobacillus*–BSA binding to BaP fit the pseudo-second-order kinetic model, whereas those of *Lactobacillus* binding to BaP fit the Elovich kinetic model. It can be presumed that BaP binding is influenced by chemisorption during the binding process. Zoghi et al. [36] found that chemisorption was also involved in patulin adsorption by NaOH-treated *L. plantarum* ATCC 8014 in apple juice by analyzing a pseudo-second-order kinetic model. Additionally, the Weber–Morris intra-particle diffusion model was used to understand the diffusion mechanism and predict the rate-limiting step of the adsorption of BaP by *Lactobacillus*–BSA. If the plot gives a straight line and passes through the origin, it means that the binding process of BaP is controlled only by intra-particle diffusion [37]. The two straight lines do not pass through the origin, thus indicating that the BaP-binding process is controlled by intra-particle diffusion and liquid membrane diffusion.

## 5. Conclusions

In this study, the combination of *Lactobacillus* and BSA enhanced the BaP-binding effect. Better stability, complete cell morphology and a desirable influence of the gastrointestinal environment were obtained in the BaP binding by *Lactobacillus*–BSA. Additionally, polysaccharides, lipids, and proteins were involved in BaP binding. The pseudo-second-order kinetic model and Freundlich isotherm model were more suitable for describing the binding behavior of *Lactobacillus*–BSA to BaP. These findings demonstrate a novel method for the combination of *Lactobacillus* and BSA for the effective removal of BaP.

## Figures and Tables

**Table 1:** The survival rate of strains in the simulated gastrointestinal tract in the presence of BSA.

Strains	Initial Concentration/(CFU/mL)	Simulated Gastric Juice		Simulated Intestinal Juice	
		Viable Count/(CFU/mL)	Survival Rate/(%)	Viable Count/(CFU/mL)	Survival Rate/(%)
121	1.14 × 10^10^	9.75 × 10^9^	85.53	9.7 × 10^8^	8.51
121-BSA		7.9 × 10^9^	69.30	1.65 × 10^9^	14.47
ML32	1.56 × 10^10^	1.2 × 10^10^	76.92	1.9 × 10^9^	12.18
ML32-BSA		1.25 × 10^10^	80.12	2.06 × 10^9^	13.20

**Table 2:** The BaP-binding ability of *Lactobacillus*–BSA in the simulated gastrointestinal tract.

Strains	Simulated Gastric Juice		Simulated Intestinal Juice	
	Cells	Cells Combined with BSA	Cells	Cells Combined with BSA
121	69.64 ± 1.24	74.17 ± 0.88 *	6.56 ± 4.68	6.33 ± 0.58
ML32	69.71 ± 2.32	72.86 ± 0.99 *	4.64 ± 3.39	16.88 ± 2.23 *

Note: * *p* < 0.05.

**Table 3:** The correlation analysis between the BaP binding percentage and surface properties of strains.

Strains	Pearson Correlation Coefficient	
	Surface Hydrophobicity	Automatic Aggregation
121	0.19	0.09
ML32	0.22	−0.13

**Table 4:** The Langmuir and Freundlich isothermal model fitting results.

Strains	Langmuir Model			Freundlich Model		
	*K_L_*	*q_max_*	*R* ^2^	*K_F_*	*n*	*R* ^2^
121	4.3167 × 10^−6^	1.0728 × 10^5^	0.9654	0.0919	0.6988	0.9975
121-BSA	7.6971 × 10^−6^	3.3348 × 10^5^	0.9560	0.9182	0.7320	0.9911
ML32	2.2721 × 10^−5^	1.5443 × 10^4^	0.9656	0.4125	1.0443	0.9661
ML32-BSA	8.2851 × 10^−6^	2.4599 × 10^5^	0.9679	0.9074	0.7865	0.9905

**Table 5:** The fitting parameters of adsorption kinetics models.

Strains	Qexp	Pseudo-First-Order Kinetic			Pseudo-Second-Order Kinetic			Elovich Model		
		*q_e_*	*K*_1_	*R* ^2^	*q_e_*	*K*_2_	*R* ^2^	*a*	*b*	*R* ^2^
121	6.0189	5.6158	0.2255	0.8793	6.3520	0.0477	0.9646	1.9525	1.1272	0.9793
121-BSA	8.3920	7.8194	0.5710	0.8262	8.2810	0.1197	0.9681	5.2774	0.8400	0.9540
ML32	6.5945	6.0094	0.3248	0.6032	6.6501	0.0684	0.8294	2.8213	1.0239	0.9417
ML32-BSA	9.0297	8.4909	0.3962	0.8432	9.2421	0.0631	0.9708	4.5667	1.2706	0.9686

## Data Availability

The data are available from the corresponding author.
